# Benefits of Newborn Screening for Vitamin D-Dependant Rickets Type 1A in a Founder Population

**DOI:** 10.3389/fendo.2022.887371

**Published:** 2022-05-06

**Authors:** Carol-Ann Fortin, Lysanne Girard, Chloé Bonenfant, Josianne Leblanc, Tania Cruz-Marino, Marie-Eve Blackburn, Mathieu Desmeules, Luigi Bouchard

**Affiliations:** ^1^ Biochemistry and Functional Genomics Department, Faculty of Medicine and Health Sciences (FMHS), Université de Sherbrooke, Sherbrooke, QC, Canada; ^2^ Department of Laboratory Medicine, Centre intégré universitaire de santé et de services sociaux (CIUSSS) du Saguenay-Lac-Saint-Jean – Hôpital de Chicoutimi, Chicoutimi, QC, Canada; ^3^ ÉCOBES, Recherche et transfert, Cégep de Jonquière, Jonquière, QC, Canada; ^4^ Medicine Department, FHMS, Université de Sherbrooke, Sherbrooke, QC, Canada; ^5^ Clinique des maladies métaboliques, CIUSSS du Saguenay-Lac-Saint-Jean – Hôpital de Chicoutimi, Chicoutimi, QC, Canada

**Keywords:** VDDR1A, newborn screening, CYP27B1, vitamin D, calcitriol, Saguenay-Lac-Saint-Jean

## Abstract

**Background:**

Vitamin D-dependant rickets type 1A (VDDR1A) is a rare autosomal recessive disorder caused by pathogenic variants in the *CYP27B1* gene. This gene is essential for vitamin D activation. Although VDDR1A is a rare condition worldwide, its prevalence is high in the Saguenay-Lac-Saint-Jean (SLSJ) region due to a founder effect. Daily intake of calcitriol before the onset of clinical manifestations can prevent them in affected children.

**Methods:**

A genetic screening test was developed and validated for the *CYP27B1* gene c.262del pathogenic variant. Newborn screening was implemented in the SLSJ region for this variant, and the feasibility and acceptability were assessed. Sixteen medical records of children affected with VDDR1A were reviewed to document the consequences of the disease at diagnosis.

**Results:**

A total of 2000 newborns were tested for VDDR1A. Most families (96.5%) accepted the genetic test. We found a carrier rate of 1/29 for the c.262delG variant in our cohort, which is suggestive of a founder effect. We identified one child affected with VDDR1A and treatment was initiated before the onset of clinical manifestations. On average, children with VDDR1A were diagnosed at 13.8 ± 5 months of age, they had a significant failure to thrive at diagnosis, among other harmful health consequences.

**Conclusion:**

Our study showed that in our population, the newborn genetic screening program is safe and feasible, it has high acceptability, and it is efficient to identify affected children. VDDR1A health consequences can be prevented by early initiation of treatment. Therefore, screening programs should be available for populations where it is deemed as beneficial from a public health perspective.

## Introduction

Vitamin D-dependent type 1A rickets (VDDR1A, MIM 264700) is an inherited autosomal recessive disorder caused by pathogenic variants within *CYP27B1* (Cytochrome P450 Subfamily 27 Family B Member 1) gene (MIM 609506), essential for vitamin D activation (1). Vitamin D is a micronutrient functioning as a hormone and essential for normal bone growth, calcium metabolism, and tissue differentiation (1, 2). The main source of vitamin D in humans is exposure to sunlight and diet, as well as dietary supplements (3). Dietary vitamin D follows a two-step sequential activation, first in the liver and then in the kidneys. The kidneys play a major role through the 1α-hydroxylation of calciferol to calcitriol; the enzyme 1α-hydroxylase is coded by the *CYP27B1* gene (3, 4). Only calcitriol can bind to the vitamin D receptor (VDR) and activate the many associated signaling pathways regulating cell proliferation, cell signaling, antioxidant effects, calcium signaling, and activation of epigenetics mechanisms among others (4).

Pathogenic variants in the *CYP27B1* gene result in low or undetectable levels of 1α-hydroxylase (2, 5–7). So far, 81 *CYP27B1* pathogenic variants have been reported in the Human Gene Mutation Database (8). Individuals affected with VDDR1A have normal or elevated levels of calciferol and collapsed levels of calcitriol. Calcitriol is regulated by parathyroid hormone (PTH), phosphorus, fibroblast growth factor 23 (FGF23), and calcitriol itself (9). Therefore, VDDR1A is also associated with increased PTH and alkaline phosphatase (ALP) but normal or decreased calcium levels (10–12).

VDDR1A is generally diagnosed between 6 to 18 months of age based on clinical manifestations such as failure to thrive, rickets, hypotonia, seizures, bone fractures, developmental delay, and enamel hypoplasia, as well as hypocalcemia, hypophosphatemia, and secondary hyperparathyroidism (12, 13). Daily intake of calcitriol and close clinical follow-up help to resolve most of the clinical manifestations, but only after around one year of treatment and depending on age at diagnosis. However, enamel hypoplasia cannot be corrected when the treatment is started after its presentation (14, 15). Neonatal molecular screening within the first two weeks after birth and calcitriol treatment of affected newborns should allow to prevent all VDDR1A consequences and normalize their overall development. Although VDDR1A treatment is fully efficient, inexpensive, available, and has very limited side effects, its neonatal screening is challenging because of the cost to test 81 pathogenic variants and its overall low prevalence worldwide (219 patients reported in the literature) (9).

Nevertheless, some regions in the world could benefit from VDDR1A neonatal screening and pave the way for larger initiatives (16). The Saguenay-Lac-Saint-Jean (SLSJ), Haute-Côte-Nord (HCN), and Charlevoix regions are characterized by an increased prevalence or frequency for VDDR1A. Other recessive disorders also show an increased prevalence in these regions of Québec, Canada (17, 18).

In brief, only the NM_000785.4(*CYP27B1*): c.262del (p.Val88Trpfs*71) pathogenic variant has been detected so far in this French-Canadian population (19). The deletion of a guanine at position 262 in exon 2 results in a frameshift and a truncated protein, which lacks the 1α-hydroxylase activity (20). In the SLSJ region, the c.262del carrier rate is estimated as 1/27 and the VDDR1A prevalence as 1/2916 (18, 19). We estimate that one affected child is born every year for a total of around 75 affected living people in the SLSJ region.

Therefore, our study aimed to 1. Demonstrate the clinical utility of VDDR1A newborn diagnosis in a founder population as a proof of concept; 2. Assess the parental acceptability for newborn screening; and 3. Measure the short-term clinical benefits of an early diagnosis on newborn development.

## Methods

### Genetic Test Development

The molecular diagnostic assay we have developed is based on nucleic acid amplification by PCR and TaqMan chemistry. It targets the pathogenic variant c.262del within the *CYP27B1* gene, which causes VDDR1A. The variant is detected by two sets of specific primers and probes we have designed and validated. The two sets of primers and TaqMan probes were directed against the pathogenic variant c.262del and independently tested to improve the analytical validity of the assay. The sequences of the probes and primers used are shown in **Supplementary Table 1**. For each amplification reaction, 1.75µL of sterile water, 3.10µL of GTXpress Master Mix, 0.15µL of TaqMan assay, and 1.2µL of samples were used. Analysis was carried out in a 96-well plate and samples were amplified on a 7500Fast Real-Time PCR thermal cycler from Applied Biosystems Inc. The amplification conditions were as follows: 1. 60°C for 1 minute with fluorescence acquisition, 2. 95°C for 20 seconds, 3. 95°C for 3 seconds, and 60°C for 30 seconds with fluorescence acquisition (repeat step 3 forty times), 4. 60°C for 1 minute with fluorescence acquisition.

### Frequent Variant Confirmation and Genetic Test Validation

To date, only the c.262del pathogenic variant described above has been identified in the SLSJ population (19). To confirm the results of the previous report, we have genotyped 13 children with VDDR1A as well as 27 parents of children with VDDR1A for whom the genotype had never been confirmed.

To ensure analytical validity of our assays, 12 samples for which we had a result of next-generation sequencing or targeted genotyping for the pathogenic variant c.262del obtained in certified clinical laboratories (either at Blueprint Genetics in Finland or GeneDx in the United States) were reanalyzed. These twelve persons were contacted by a member of the Medical Genetics Department of the CIUSSS-SLSJ and invited to participate to our technical validation. A self-collection kit (oral cells taken with swabs) was sent to each of the participants. Analysis of the pathogenic variant c.262del was performed as described above.

### Implantation of Newborn Screening in SLSJ

To assess the clinical validity and feasibility of a newborn screening program, we implemented the test in all hospitals with birthing centers in CIUSSS-SLSJ, namely the hospitals of Chicoutimi, Alma, Roberval, and Dolbeau. At birth, authorization to contact one of the parents was obtained by a clinical nurse so that a member of our research team could contact the potential participants to explain the research project objectives, the disease characteristics, and its consequences, and to obtain a consent to participate. Given the current context of the COVID-19 pandemic, meetings with parents were conducted by phone and a member of the research staff obtained verbal consent during the phone call. For those families for whom we obtained consent, a clinical nurse collected a buccal cell sample using cotton swabs, at approximately 36 hours after birth. Two samples were collected, one from each inner cheek mucosa. Analysis of the pathogenic variant c.262del was performed as described above. Results for unaffected children were mailed to parents. Parents of affected children were contacted by a pediatrician to initiate the clinical follow-up according to the parent’s consent and results were confirmed in a certified clinical laboratory.

### Acceptability Survey

Upon receipt of the result, parents were invited to complete an online anonymous survey. Age, gender, education level, and parental origin were collected. Parents were also asked to answer questions to assess their basic knowledge of human genetics. Finally, the parents answered questions that allowed us to evaluate their level of satisfaction related to the research project and its acceptability.

### Medical Records Review

Medical records of 16 affected children were reviewed to document the health consequences of a delayed diagnosis during the first 5-years of life. Of these, 15 had been diagnosed based on the onset of VDDR1A clinical manifestations, and one at-risk child was diagnosed at birth through genetic molecular testing since a first-degree relative was affected. The length and weight at birth and diagnosis, the repercussions on the child’s development, the medical treatments they received, the number of medical visits as well as their metabolic bone profile at diagnosis (PTH, total and measured ionized calcium, ALP, inorganic phosphorus, 25(OH) and 1,25(OH)_2_ vitamin D levels were collected from medical records. For the child with an early diagnosis, the metabolic bone profile was also collected and compared with that of affected children with a delayed diagnosis, to assess how early diagnosis and treatment initiation could impact the health profile.

## Result

### Observed Frequency of the c.262del (p.Val88Trpfs*71) Variant

A total of 13 affected children from the SLSJ region and their parents were recruited and genotyped for the c.262del pathogenic variant. The presumptive diagnosis was based on clinical manifestations and metabolic bone blood profile including assessment of vitamin D levels and its precursors. All children were found homozygous for the c.262del pathogenic variant, which allowed the establishment of the confirmatory diagnosis. Twenty-seven parents also provided DNA samples. They were all found heterozygous for the same pathogenic variant, which is concordant with an autosomal recessive mode of transmission. These results suggest that this could be a founder variant in this population.

### Validation of the Genetic Testing Assay

For this project, we have developed and validated a genetic testing assay for the c.262del pathogenic variant. We had access to 12 samples for validation of our assays: two individuals were heterozygous for the pathogenic variant and the wildtype allele, three individuals were homozygous for the c.262del pathogenic variant, and seven individuals were homozygous for the wildtype allele. These samples had been previously genotyped at certified clinical laboratories. All our results were in agreement with those obtained at the reference certified clinical laboratories (**Table 1**).

**Table 1:** Assay validation for the newborn diagnosis of VDDR1A by searching for the pathogenic variant c.262del.

ID	Results ÉPIMET	Results certified clinical laboratories
1	wt/wt	wt/wt
2	wt/wt	wt/wt
3	wt/wt	wt/wt
4	wt/wt	wt/wt
5	wt/wt	wt/wt
6	wt/wt	wt/wt
7	wt/wt	wt/wt
8	wt/c.262del	wt/c.262del
9	wt/c.262del	wt/c.262del
10	c.262del/c.262del	c.262del/c.262del
11	c.262del/c.262del	c.262del/c.262del
12	c.262del/c.262del	c.262del/c.262del

The results of genotyping performed in the ÉPIMET laboratory at the Chicoutimi Hospital are in agreement with the results of genotyping performed in the certified clinical laboratories.

### VDDR1A Newborn Screening Program Feasibility

In May 2020, we have launched a clinical study to assess the feasibility and acceptability of a VDDR1A screening program in the SLSJ region and to address issues related to its founder effect. Our *a priori* goal of testing 2,000 newborns had been completed by July 2021. On average, parents were informed of the result 14 days (+/- 7) after buccal cells were collected. A total of 70 newborns were heterozygous (carriers) for the c.262del pathogenic variant for an estimated carrier rate of 1/29. Carrier children were classified as unaffected for the pathogenic variant c.262del and the carrier status of the children was not reported to the parents. In addition, one affected child (homozygous for the pathogenic variant c.262del) was identified. Treatment was initiated within two weeks following the molecular diagnosis.

### Characteristics of Survey Participants

**Figure 1:**
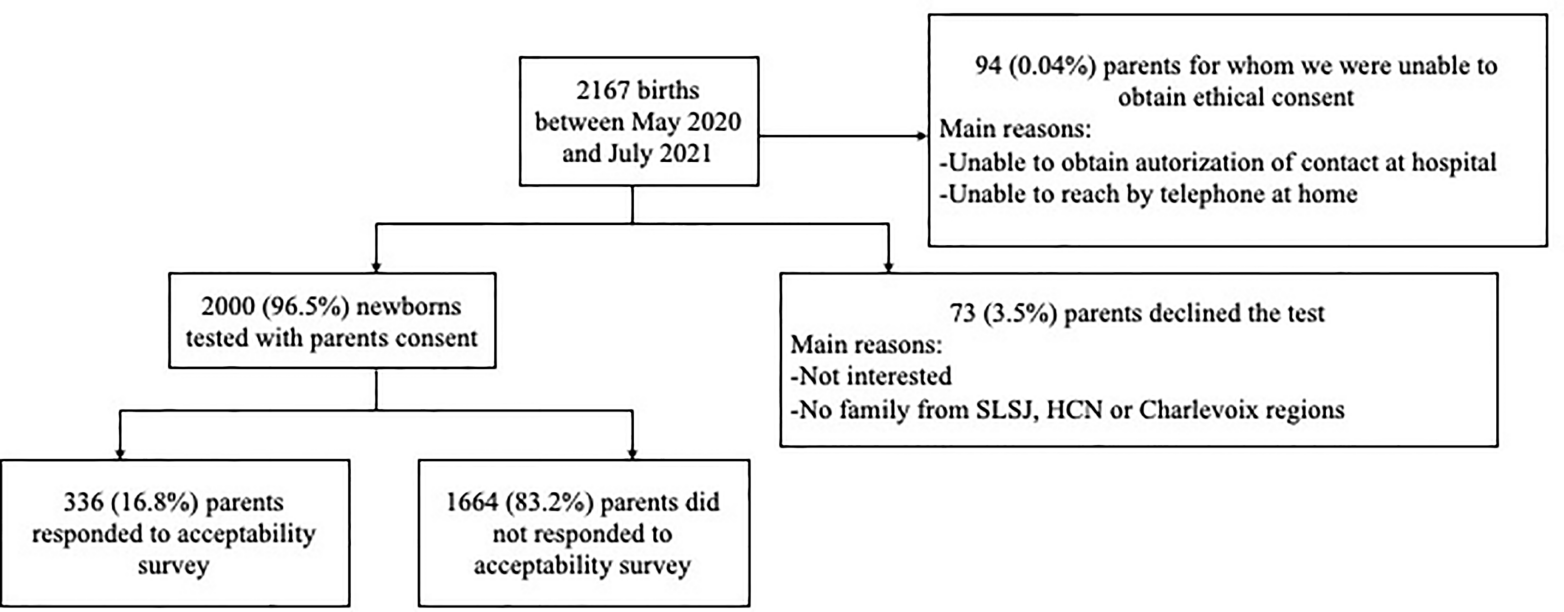
Flowchart of newborn screening for VDDR1A.

To assess the acceptability of a newborn screening program for VDDR1A, the 2,000 families who participated in our project were asked to complete an online anonymized questionnaire. A total of 336 participants completed the questionnaire (**Figure 1**). The characteristics of these participants were summarized in **Table 2**. In brief, 65.1% of the participants were between 26 and 35 years old of age and 64.3% identified themselves as females. Thirty-six percent (36.0%) completed a university degree, 27.7% completed college, 24.4% completed high school and 11.9% of the responders did not complete high school. Most mothers (83.0%) reported they had at least one grandparent from SLSJ, HCN, or Charlevoix regions, while this antecedent was present in 80.1% of the fathers.

**Table 2:** Characteristics of the participants that completed the survey on VDDR1A newborn screening acceptability.

Characteristics n=336	n (%)
**Age**	
Less than 18 years	1 (0.3%)
18 - 25 years	62 (18.5%)
26 - 30 years	111 (33.0%)
31-35 years	108 (32.1%)
36-40 years	44 (13.1%)
41-45 years	9 (2.7%)
More than 45 years	1 (0.3%)
**Sex**	
Male	80 (23.8%)
Female	216 (64.3%)
Other	40 (11.9%)
**Highest level of education completed**	
High school not completed	40 (11.9%)
High school completed	82 (24.4%)
College completed	93 (27.7%)
University completed	121 (36.0%)
**Having at least one grandparent from SLSJ, HCN, or Charlevoix**	
**Maternal ancestry**	
Yes	279 (83.0%)
No	53 (15.8%)
Don’t know	4 (1.2%)
**Paternal ancestry**	
Yes	269 (80.1%)
No	10 (3.0%)
Don’t know	5 (1.5%)

### VDDR1A Newborn Screening Acceptability

In addition to the 2,000 families who agreed to participate in the research project, 73 families declined the test, and we were unable to reach the family or to obtain authorization to be contacted for another 94 families. The overall acceptability rate reached 96.5% (**Figure 1**).

Information allowing to estimate the acceptability were summarised in **Table 3**. Briefly, 93.7% of the participants report that newborn VDDR1A screening is essential or useful. Moreover, 96.1% of them would probably or certainly recommend the screening test to someone they know who is planning pregnancy or is pregnant. Finally, 97.0% of respondents would agree that the screening test should be offered to all children with at least one grandparent from SLSJ, HCN, or Charlevoix.

**Table 3:** Distribution of the answer provided on the acceptability survey.

Answers (n=336)	n (%)
**“In your opinion, is getting tested for vitamin-dependent rickets…?”**	
Essential	107 (31.8%)
Useful	208 (61.9%)
Of little use	8 (2.4%)
Useless	3 (0.9%)
Don’t know	9 (2.7%)
**“Would you recommend the screening test to someone you know who is going to have a baby?”**	
Certainly	247 (73.5%)
Probably	76 (22.6%)
Probably not	3 (0.9%)
Certainly not	1 (0.3%)
Don’t know	8 (2.4%)
**“Would you agree that the screening test be offered to all children with at least one grandparent from SLSJ, HCN, or Charlevoix?”**	
Totally agree	277 (82.4%)
Rather agree	49 (14.6%)
Little agree	3 (0.9%)
Not agree at all	0 (0.0%)
Don’t know	7 (2.1%)

### Review of Medical Records of Affected Children

**Figure 2:**
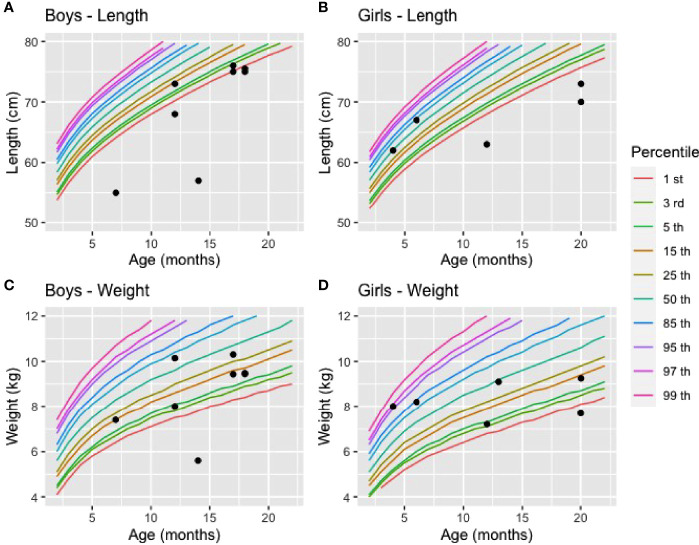
Length and weight of affected boys and girls at diagnosis compared to reference values **(A)** Length of boys (n=8), **(B)** Length of girls (n = 5), **(C)** Weight of boys (n = 7), **(D)** Weight of girls (n = 6). The reference values for weight and length in percentile were based on the World Health Organisation charts (2021).

Data from the first 5 years of life were extracted from the medical records of 16 affected children born between 1990 and 2020. Of those, 15 were diagnosed after the onset of clinical manifestations, and one child was diagnosed before the onset of the disease. Observations for those with a delayed diagnosis were summarized in **Table 4**. These children received VDDR1A diagnosis on average at 13.8 months +/-5 months (range: 4.0 to 20.0 months) after clinical suspicion based on clinical features consistent with VDDR1A. At birth, the children had a mean weight of 3310.9g (2555.0-3825.0) and a length of 49.2cm (45.0-52.0). All children were within normal ranges when compared to the World Health Organization (WHO) weight and length charts (weight: 4.0-86.9 percentile; length: 1.3-86.9 percentile). At diagnosis, the children had a mean weight of 8.5kg (5.6-10.3) and they were 68.4 cm (55.0-76.0) tall. Although the children’s weight at diagnosis remained within the normal range for all but two boys and girls, their length was below the first percentile for five boys out of eight and for three girls out of five (**Figure 2**).

**Table 4:** Characteristics of VDDR1A affected individuals for which medical records were reviewed.

Clinical data (n=15)	n	Mean ± SD	Range
**At birth**			
Weight (g)	14	3310.9 ± 382.6	2555.0 – 3825.0
Length (cm)	14	49.2 ± 2.3	45.0 – 52.0
Weight for age (percentile)	14	51.3 ± 25.7	4.0 – 86.9
Length for age (percentile)	14	47.0 ± 32.0	1.3 – 86.9
Sex			
Female *n* (%)	7	7 (47%)	
Male *n* (%)	8	8 (53%)	
**At diagnosis**			
Age (months)	15	13.8 ± 5.0	4.0 – 20.0
Weight (kg)	14	8.5 ± 1.3	5.6 – 10.3
Length (cm)	13	68.4 ± 7.2	55.0 – 76.0
Weight for age(percentile)	14	28.3 ± 32.4	0 – 96.1
Length for age(percentile)	13	10.4 ± 22.3	0 – 70.0

SD, Standard deviation.

One child’s diagnosis was assessed at two weeks of life by genetic diagnosis because a first-degree relative had a history of VDDR1A whereas the other 15 children were diagnosed by the development of clinical manifestations at a mean age of 13.8 months. For those with a delayed diagnosis, eight children were diagnosed following a consultation with a pediatrician, while the other seven were diagnosed following an emergency room visit. The main reasons for diagnosis were developmental delay (n=8), failure to thrive (n=3), multiple fractures (n=2) and seizures (n=1). Out of 16 individuals, seven cases had to be hospitalized following the diagnosis (**Table 5**).

**Table 5:** Clinical manifestations and medical history at diagnosis.

Participant	Age at diagnosis (months)	Pediatric investigation	Investigation in the emergency room	Failure to thrive	Developmental delay	Hospitalization	Multiple fractures	Seizures	Newborn genetic diagnosis	Family history of VDDR1A
1	0.4								√	1^st^ degree
2	4	√								2^nd^ degree
3	6	√								1^st^ degree
4	7		√			√				None
5	12		√							None
6	12	√								None
7	12		√		√	√	√			None
8	13	√			√					None
9	14	√			√					4^th^ degree
10	17		√		√	√				None
11	17	√		√	√					None
12	17		√		√	√		√		4^th^ degree
13	18	√			√					4^th^ degree
14	18	√				√				None
15	20		√	√		√				None
16	20		√	√	√	√	√			None
	Total	8	7	3	8	7	2	1	1	

### Metabolic Bone Profile in Affected Individuals

Metabolic bone profiles for 15 affected individuals with a delayed diagnosis (13.8 monrths on average) and 1 individual with an early diagnosis were also available (**Table 6**). For the former, all values were well outside than the normal references, except for 25(OH) vitamin D, as expected. For the latter, only a slight dysregulation of his/her metabolic bone profile was reported. This child benefited from calcitriol treatment starting at diagnosis (1 month). His/her metabolic bone profile was very close to the reference values at 14 months compared to the metabolic bone profile of children who received their diagnosis around the same age (13.8 months on average).

**Table 6:** Metabolic bone blood profile of affected individuals at the time of late and early diagnosis.

	n	Late diagnosis mean ± SD	Early diagnosis (n=1) mean		Reference values
Age (months)	15	13.8 ± 5	1	14	
PTH (pmol/L)	12	64.4 ± 25.2	8.3	3.7	1.30-9.30
Total calcium (mmol/L)	14	1.7 ± 0.2	2.64	2.61	2.22-2.70
Measured ionized calcium (mmol/L)	15	0.9 ± 0.1	NA	1.4	1.15-1.35
ALP (U/L)	15	2215.9 ± 1052.4	346	333	40-160
Inorganic phosphorus (mmol/L)	15	1.1 ± 0.2	2.31	1.86	1.45-2.10
Vitamin D 25(OH) (nmol/L)	11	104.8 ± 35.0	197.6	101.2	75-250
Vitamin D 1-25 (OH)_2_ (pmol/L)	11	4.6 ± 2.6	NA	NA	78-125

For the child with an early diagnosis, the metabolic bone profile was also collected at 14 months and compared with that of affected children with a delayed diagnosis, to assess how early diagnosis and treatment initiation could impact the health profile.

SD, Standard deviation; ALP, Alkaline phosphatase; PTH, Parathyroid hormone.

### Growth Between Birth and Diagnosis for Affected Individuals

**Figure 3:**
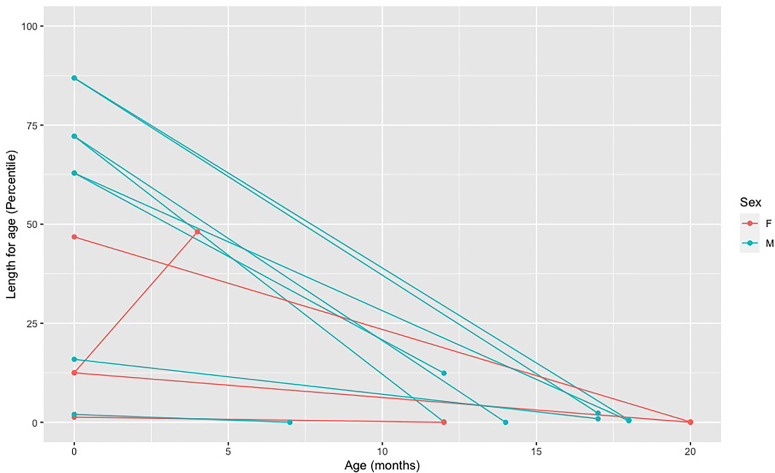
Decreased in length percentile from birth to diagnosis in affected individuals. Each point represents a length measure in percentile taken at a different age. The first dot (far left) represents the length at birth and the second dot represents the length at the age of diagnosis. The two dots of each individual are connected by a line.

**Figure 4:**
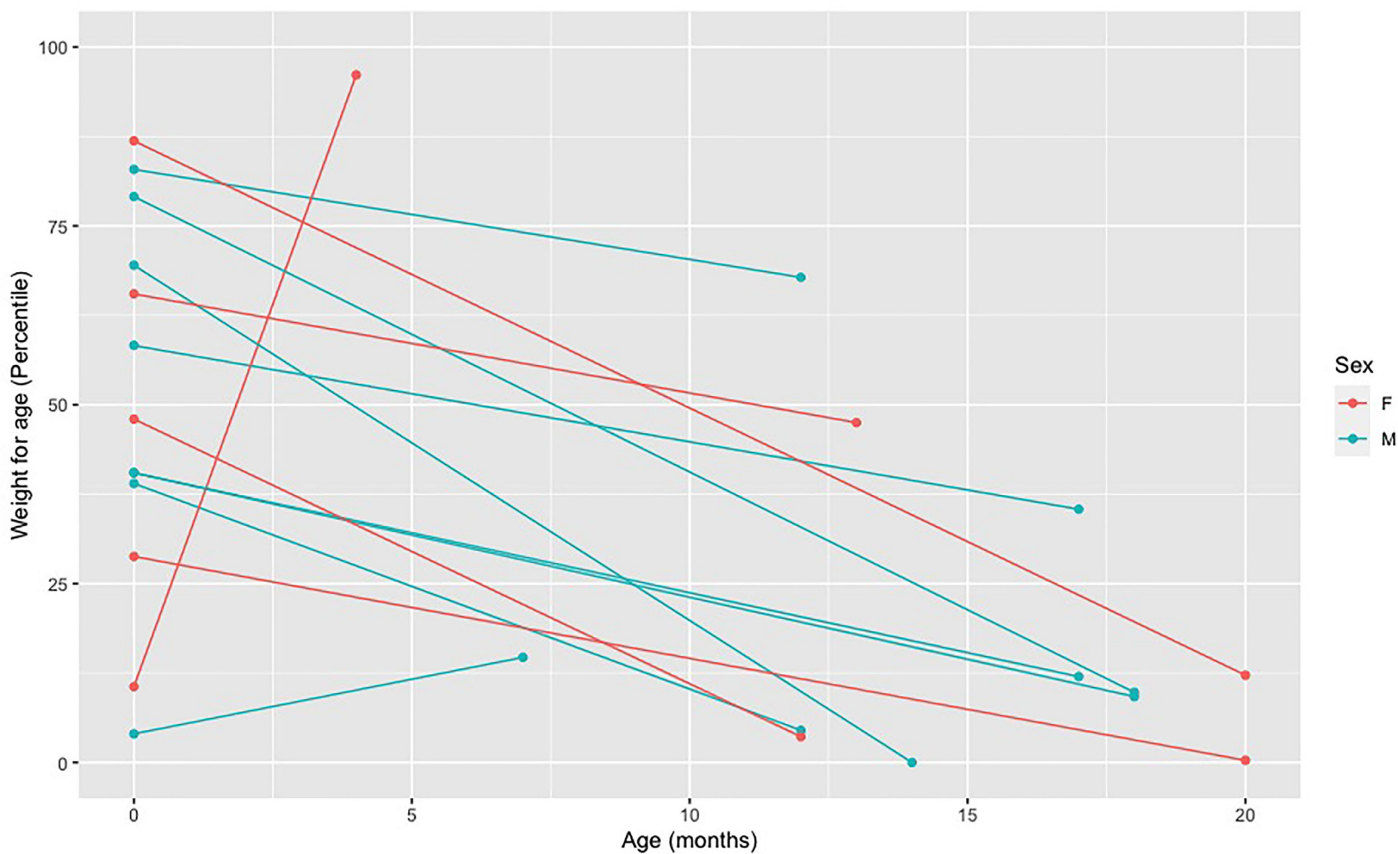
Decreased in weight percentile from birth to diagnosis in affected individuals. Each point represents a weight measure in percentile taken at a different age. The first dot (far left) represents the weight at birth and the second dot represents the weight at the age of diagnosis. The two dots of each individual are connected by a line.

A length/weight for age below the fifth percentile and a weight deceleration crossing two major percentile lines are frequently used criteria to indicate a failure to thrive and monitor growth (21). Active vitamin D deficiency in children with VDDR1A appears to affect child’s length more than weight. Overall, we have observed a marked decrease in length percentile from birth to diagnosis for all but one child. This child was diagnosed at four months of age based on a family history of VDDR1A and was still free of apparent clinical manifestations of the disease (**Figure 3**). For weight, we have observed a slight decrease in percentile for all but two children, who were diagnosed at four and seven months of age (**Figure 4**).

## Discussion

Although VDDR1A remains a rare disease worldwide, its frequency is higher in the SLSJ region, probably due to a founder effect (18, 19). The importance of early diagnosis and treatment for VDDR1A have been well documented in the past and the present study (9, 22–24). These studies highlighted the importance of offering an early diagnosis to individuals affected with VDDR1A to prevent related harmful health consequences (13, 25). However, establishing the diagnosis before the onset of manifestations remains challenging due to the multiple pathogenic variants that can cause VDDR1A and because the family history of the disease is most often unknown for autosomal recessive disorders such as VDDR1A. In this study, we showed that a newborn genetic screening program for VDDR1A is safe, feasible, has high acceptability rates, and is efficient to identify affected children in a population where a variant is known to be frequent. This paved the way for early treatment initiation and prevention of the related harmful health consequences.

First, we were able to confirm the c.262del genotype of children affected with VDDR1A and their parents. To our knowledge, only two other variants have been reported in the French-Canadian population. The first variant was reported in only one individual in 1998 by Yoshida and collaborators: a 7-bp insertion in exon 8 that causes a frameshift at codon 443 and premature truncation of the protein (20). The other variant has also been reported in only one individual in 1998 by Wang and collaborators: c.1166G>A (p.Arg389His) (5). These two pathogenetic variants were not tested per se in the current study, but all results are concordant with a low to a very low carrier rate in our population. We were also able to confirm the carrier rate of c.262del (1/27), which had been estimated in 1991 by De Braekeleer and Larochelle using the Hardy-Weinberg law, based on the number of births of children affected with VDDR1A (19).

Many studies have also described frequent misdiagnosis of VDDR1A, mainly in cases of nutritional rickets, renal tubular acidosis, and hypophosphatemic rickets. In these studies, the mean age at onset was 12 (9-18) months whereas the age at diagnosis was 20 (13-31) months. These data were obtained by retrieving results from 36 studies and analyzing a total of 156 patients. (9) In our cohort, the age at diagnosis was 13.8 (4-20) months. This relative precocity of diagnosis could be explained by the high prevalence of VDDR1A in SLSJ and the potential related founder effect. Only one other region has been reported to have a high frequency of VDDR1A caused by the pathogenic variant c.195+2T>G in intron 1 of the *CYP27B1* gene, suggesting a founder effect in Turkish (23, 26–28). The mean age at diagnosis for their cohort of 11 patients with VDDR1A was 13.1 (6-36) months, similar to our cohort (23). Misdiagnosis can lead to delayed diagnosis and treatment (>1 year) which can result in permanent deformities and short stature (9).

Our study also resulted in the early diagnosis of a child with VDDR1A, long before the onset of manifestations. To our knowledge, only one other affected individual has been diagnosed this early in this population and the absence of any VDDR1A-related features. This child had a first-degree relative affected with VDDR1A. At diagnosis (1 month), this child had a normal serum level of calcium, phosphorus, ALP, and 25(OH) vitamin D, but a low level of 1,25(OH)_2_ vitamin D. His/her diagnosis was later confirmed with the identification of a homozygous c.262del genotype (referred to as 958delG) (13). These results are concordant with our findings, but the 1,25(OH)_2_ D serum levels were not quantified for the child identified in our study.

Kaygusuz and collaborators have demonstrated poor final length and osteopenia in noncompliant patients suggesting that early initiation of treatment and good compliance are essential in achieving normal length and bone mineral density (29). Our results showed that a delayed diagnosis would have a greater effect on children’s length, but also on weight. Further longitudinal studies could evaluate whether the children’s growth normalizes completely and evaluate compliance to treatment.

The WHO bases its recommendations for newborn screening on the 10 criteria established by Wilson and Jungner in 1968 (30, 31). In short, the condition must be significant and there must be an effective treatment to prevent the disease, decrease the progression or ideally cure it. There must be a clear screening plan that is effective, acceptable, and affordable. In Quebec, the National Screening Committee has issued recommendations to assess the appropriateness of adding diseases to the Quebec Newborn Screening Program. These recommendations, based on the 14 criteria issued by the National Screening Committee in the United Kingdom, are divided into four categories concerning the condition, the screening test, the treatment, and the program (32). Briefly, the disease to be screened must be an important and well-characterized health problem. The screening test must be simple, safe, accurate, valid, and acceptable to the population. The treatment must be effective, have guidelines for patients to be treated and the benefits of early treatment must be well-identified. Finally, the program reduces mortality and/or morbidity, is clinically, socially, and ethically acceptable to health care professionals and the public, and the benefits of the program are judged to outweigh the physical and psychological harm caused by tests, clinical procedures, and treatments (33). The present study demonstrated that newborn screening for VDDR1A for the SLSJ population meets all of these criteria.

The founder effect reported in SLSJ Region also extends to Charlevoix and HCN regions, whereas the VDDR1A carrier frequency has not been specifically assessed in these regions so far (19). Although expected, our study was limited by the number of patients with VDDR1A diagnosed at birth through genetic testing with only one affected child detected. Indeed, it must be considered that despite its high carrier rate in the SLSJ, this condition’s prevalence is approximately 1 affected child per 2916 births, which represents 1 affected childbirth per year in SLSJ. Accordingly, the long-term benefits of a diagnosis obtained at birth will have to be assessed in further studies. Our study was also limited by the number of affected children to whom we had access to validate our assay and document the impacts of VDDR1A during the first 5 years of life. Such numbers of cases (and carrier parents) for a rare disease remains nevertheless noteworthy.

This study allowed to offer for the first time a newborn screening program for VDDR1A. We were able to assess the relevance of offering VDDR1A genetic newborn screening in the SLSJ population based on the criteria of the National Screening Committee in Quebec. We conclude that the disease is prevalent in the SLSJ population (it is estimated that approximately 1 child is born with the disease per year), that early treatment of the disease prevents consequences for affected children, and that parental acceptability of a screening program is very high. Feasibility is also very high as commitment from the physician and nurses has been easy to obtain considering that VDDR1A affects children, that the treatment has no side effects, and that all the harmful consequences will be prevented if it initiates within the first week of life. The test we have developed is safe, accurate, and affordable. VDDR1A newborn screening should be available to any population with an increased prevalence of this condition, who would benefit from this public health initiative.

## Data Availability Statement

The original contributions presented in the study are included in the article/supplementary material, further inquiries can be directed to the corresponding authors.

## Ethics Statement

The studies involving human participants were reviewed and approved by Comité d’éthique de la recherche, Centre intégré universitaire de santé et services sociaux CIUSSS du Saguenay—Lac-Saint-Jean. Written informed consent to participate in this study was provided by the participants’ legal guardian/next of kin.

## Author Contributions

LB designed the study with the contribution of MD, TC-M, JL, and C-AF; LG and CB performed data collection with C-AF; C-AF performed medical records review and performed statistical analyses, M-EB designed the acceptability survey and supervised the data collection, C-AF wrote the manuscript with the collaboration of LB. LB supervised all steps of the study. All authors have contributed to this work, revised, and approved the final manuscript.

## Funding

C-AF was supported by a master’s research award from the Faculty of Medicine and Health Sciences (FMHS) of Université de Sherbrooke and *Centre de recherche Charles-Le Moyne – Saguenay–Lac-Saint-Jean*. This study was supported by Fondation du Grand défi Pierre Lavoie and Centre intégré universitaire de santé et de services sociaux du Saguenay-Lac-Saint-Jean. LB is a senior Scholar from the Fonds de la recherche du Québec en santé.

## Conflict of Interest

The authors declare that the research was conducted in the absence of any commercial or financial relationships that could be construed as a potential conflict of interest.

## Publisher’s Note

All claims expressed in this article are solely those of the authors and do not necessarily represent those of their affiliated organizations, or those of the publisher, the editors and the reviewers. Any product that may be evaluated in this article, or claim that may be made by its manufacturer, is not guaranteed or endorsed by the publisher.

## Supplementary Material

Supplementary Table 1 TaqMan primers and probes used to detect *CYP27B1* c.262delG variant.

## Abbreviations

ALP, Alkaline Phosphatase; CYP27B1, Cytochrome P450 Subfamily 27 Family B Member 1; FGF23, Fibroblast Growth Factor 23; HCN, Haute-Côte-Nord; PTH, Parathyroid hormone; SLSJ, Saguenay-Lac-Saint-Jean; VDR, Vitamin D Receptor; VDDR1A, Vitamin D-Dependant Rickets Type 1A; WHO, World Health Organization.
