# Supplementary material for: Benefits of Newborn Screening for Vitamin D-Dependant Rickets Type 1A in a Founder Population
Source: Front Endocrinol (Lausanne). 2022 May 6;13:887371. doi: 10.3389/fendo.2022.887371 (PMC9120640; doi:10.3389/fendo.2022.887371)
Supplement: Supplementary Table 1 — TaqMan primers and probes used to detect CYP27B1 c.262delG variant. [file Table_1.docx]

**Supplementary table 1: TaqMan primers and probes used to detect *CYP27B1* c.262delG variant**

| Assay name: CYP27B1_c262d3*  Assay ID: ANAAPK6 | *CYP27B1* c.262delG - p.Val88Trpfs*71 |
| --- | --- |
| Forward primer | TCGCAGCAGCTCCTCGA |
| Reverse primer | CTAGCCAGCTTTGGGACAGTG |
| Probe 1 | CAGCCACGTACACGG |
| Probe 2 | CAGCCAGTACACGGT |
| Amplicon length (bp): 69 | |

| Assay name: CYP27B1_c262d4*  Assay ID: ANCFH63 | *CYP27B1* c.262delG - p.Val88Trpfs*71 |
| --- | --- |
| Forward primer | CTGCAGCGCTCGGGC |
| Reverse primer | CGCCGCGCACTTCG |
| Probe 1 | GCCACGTACACGGT |
| Probe 2 | CAGCCAGTACACGGT |
| Amplicon length (bp): 123 | |
